# Nasal Septal Deviation Classifications, Including the Mladina System, and Their Craniofacial Correlates: A Scoping Review

**DOI:** 10.3390/jcm15134853

**Published:** 2026-06-23

**Authors:** Rafał Nowak, Filip Bliźniak, Karolina Lubecka, Joanna Wołoszyn, Mateusz Kęska, Wojciech Macek, Maciej Chęciński, Maciej Sikora

**Affiliations:** 1 Department of Otolaryngology and Maxillofacial Surgery, Institute of Medical Science, University of Zielona Góra, Ul. Zyty 28, 65-046 Zielona Gora, Poland; 2 Clinical Department of Maxillofacial, Oncological and Reconstructive Surgery, 4th Military Clinical Hospital, Ul. Rudolfa Weigla 5, 50-981 Wroclaw, Poland; 3 Department of Maxillofacial Surgery, Rydygier Hospital, Osiedle Złotej Jesieni 1, 31-826 Krakow, Poland; fblizniak@gmail.com; 4 Department of Oral Surgery, Preventive Medicine Center, Komorowskiego 12, 30-106 Krakow, Poland; lubeckarolina@gmail.com (K.L.); ld.wojciech.macek@wp.pl (W.M.); 5 Dental Clinic Outpatient Care Center, University Dental Clinic in Kraków, Montelupich 4, 31-155 Krakow, Poland; joannawoloszyn234@gmail.com; 6 Faculty of Medicine, Medical College, Jagiellonian University, Świętej Anny 12, 33-332 Kraków, Poland; matteuszkeska@gmail.com; 7 National Medical Institute of the Ministry of the Interior and Administration, Wołoska 137 Str., 02-507 Warsaw, Poland; maciej.checinski@pimmswia.gov.pl (M.C.); sikora-maciej@wp.pl (M.S.); 8 Department of Maxillofacial Surgery, Hospital of the Ministry of Interior, Wojska Polskiego 51, 25-375 Kielce, Poland; 9 Department of Biochemistry and Medical Chemistry, Pomeranian Medical University, Powstańców Wielkopolskich 72, 70-111 Szczecin, Poland

**Keywords:** cone-beam computed tomography, facial asymmetry, malocclusion, mandible, maxilla, nasal obstruction, nasal septum, tomography, X-ray computed

## Abstract

**Background/Objectives:** Nasal septal deviation (NSD) is a common anatomical condition that may influence nasal airflow and has been proposed as a potential factor associated with craniofacial growth and morphology. However, available studies use heterogeneous classifications and measurement methods, including the Mladina classification, angular parameters, volumetric assessment, CT, CBCT, and cephalometric analyses. This scoping review aimed to map and synthesize the available evidence on the relationship between NSD classifications, including the Mladina system, and craniofacial morphological correlates. **Methods:** This scoping review was conducted according to Joanna Briggs Institute methodology for scoping reviews and reported in accordance with the PRISMA-ScR checklist. The protocol was prospectively registered in the Open Science Framework. A broad literature search was performed in PubMed, Embase, Cochrane Library, BASE, and Google Scholar, without restrictions on publication date or language. Eligible studies included clinical or academic investigations assessing NSD using a defined classification or quantitative parameters and relating it to craniofacial, maxillary, mandibular, dentofacial, or asymmetry-related outcomes. **Results:** From 715 identified records, 387 remained after deduplication, and 6 studies met the inclusion criteria. The included studies varied substantially in sample size, imaging modality, NSD assessment method, and outcome domains. The most consistent findings suggested associations between NSD and localized or transverse nasomaxillary changes, particularly involving the palate, maxilla, nasal floor, and dentoalveolar region. Evidence regarding global facial asymmetry, basic maxillomandibular dimensions, and malocclusion was limited and inconsistent. Studies using the Mladina classification did not provide uniform conclusions across outcome domains. **Conclusions:** Current evidence does not support NSD as a uniform marker of global craniofacial morphology abnormalities. NSD appears more plausibly associated with selected local and transverse nasomaxillary features than with overall facial asymmetry. Future studies should combine standardized NSD classifications, especially the Mladina system, with precise three-dimensional craniofacial assessment in homogeneous populations.

## 1. Introduction

### 1.1. Background

The nasal septum is a central anatomical element of the facial skeleton, separating the nasal cavity into two halves and participating in the regulation of airflow. During development, it plays a significant role in shaping the structures of the midface, and its growth and remodeling are closely related to the surrounding bones of the maxilla, palate, and skull base [1,2]. The importance of the nasal septum as a potential growth center for the facial skeleton has been emphasized in the literature for years, particularly in the context of experimental studies and clinical observations of abnormalities in its structure [3,4].

Nasal septal deviation (NSD) is one of the most common anatomical abnormalities of the nose and is found in a significant percentage of the general population. It can lead to nasal obstruction, chronic mouth breathing, and secondary functional changes in the stomatognathic system. NSD is considered not only an otolaryngological problem but also a potential factor influencing the development and morphology of maxillofacial structures [5,6,7].

Many classifications have been developed for the assessment of NSD, always aiming to systematize the morphology and determine the degree of deformity. These include classifications based on the location of the deviation (e.g., anterior, posterior), as well as classifications that take into account the presence of ridges, spines, or complex spatial deformities [8,9]. One of the most commonly used and best described is the Mladina classification, which describes seven types of NSD based on their anatomical characteristics. Types I–VI are specific, morphologically defined patterns of deviation (e.g., slight anterior deviations, pronounced inferior or posterior deformities, and the presence of ridges and spines), while type VII represents a combination of several coexisting deformities. This classification has been widely used in clinical practice, particularly in otorhinolaryngology, and in recent years has also been used in studies examining the potential impact of NSD on the development of craniofacial structures [10,11]. This means that it can also be really useful in maxillofacial surgery, specifically in orthognathic surgeries.

It should also be emphasized that not all studies use a standardized classification system. Some authors rely on angular measurements (e.g., the angle of the septum deviation from the midline), while others use linear parameters or volumetric indices calculated from computed tomography (CT) or cone-beam computed tomography (CBCT) images. The lack of standardization in defining and grading NSD complicates the comparison of study results and the unambiguous interpretation of potential relationships between septal deviation and craniofacial morphology [9,12].

### 1.2. Rationale

However, previous studies on the relationship between NSD and craniofacial morphology have yielded inconsistent results. Some authors indicate a significant relationship between the degree of septal deviation and maxillary transverse asymmetry, palatal depth, or the presence of malocclusion [12,13,14]. Other studies do not confirm such a relationship, particularly in relation to global facial asymmetry or basic jaw dimensions. Furthermore, the methods used to assess NSD are very different from each other. Methods for assessing bone structures also include traditional two-dimensional cephalometry, CT, and CBCT, which makes direct comparison of results difficult [12,15,16].

The lack of methodological consistency and discrepancies in reported correlations indicate the need to organize the available data. To date, there has been no comprehensive study systematically compiling the results of studies examining the relationship between NSD and craniofacial skeletal morphology in the human population, taking into account various imaging methods and the classification of NSDs. It is essential to map the existing literature and identify specific gaps and inconsistencies. This will provide direction for future research and establish specific goals that can be achieved through, for example, randomized controlled trials and then even perhaps meta-analyses.

### 1.3. Objectives

The aim of this scoping review is therefore to map and synthesize the available scientific evidence regarding the relationship between NSD and craniofacial morphological parameters, including maxillary and mandibular dimensions, facial asymmetry, and occlusal characteristics. The obtained results aim to define the current state of knowledge, identify areas of inconsistency, and identify research gaps requiring further analyses.

## 2. Materials and Methods

This review was conducted as a scoping study in accordance with the methodological guidance proposed by the Joanna Briggs Institute (JBI) and reported following the PRISMA-ScR (Preferred Reporting Items for Systematic Reviews and Meta-Analyses extension for Scoping Reviews) checklist [17,18,19] (Supplementary Materials). It was prepared according to the following scheme: (1) defining inclusion and exclusion criteria, (2) developing a search strategy and adapting queries to the specifics of individual databases and search engines, (3) searching leading bibliographic databases and available grey literature sources, (4) deduplication of identified records, (5) selecting studies that met the established criteria, (6) extracting and organizing data from included studies, (7) synthesizing the results in tabular and descriptive formats, and (8) presenting the main observations and conclusions resulting from the mapping of available evidence. This scoping review was prospectively registered in the Open Science Framework (OSF) on 19 February 2026 (osf.io/tsez2) [20].

Inclusion and exclusion criteria were developed according to the PCC(COSTL) approach and were determined before the literature search began (Table 1) [21]. Studies including patients diagnosed with NSD, regardless of age, were included in the review. Studies exclusively focusing on patients with genetic syndromes or congenital craniofacial defects (e.g., craniofacial syndromes) were excluded, as were studies using animal models, cadaveric materials, and in vitro studies. Publications assessing the relationship between NSD and craniofacial morphology, specifically maxillary, mandibular, and palate parameters, facial asymmetry, or malocclusion were eligible for analysis. Publications regarding sinonasal conditions affecting craniofacial morphology were excluded. NSD was required to be assessed using a specific classification system (e.g., Mladina classification) or measurable quantitative parameters (angular, linear, or volumetric). Studies that examined NSD solely in the context of ENT symptoms (e.g., nasal obstruction, sinusitis) without assessing skeletal or dentofacial parameters were excluded. Studies conducted in clinical or academic settings using radiological imaging methods such as computed tomography (CT), cone beam computed tomography (CBCT), or cephalometry were included. Studies that were purely technical or simulation-based and not based on clinical data were excluded. All study designs reporting original data were eligible for inclusion, including observational studies (cross-sectional, cohort, case–control), case series, case reports, and academic theses or dissertations. No restriction was applied regarding prospective or retrospective design. Narrative reviews, commentaries, letters to the editor, and conference abstracts without accessible full text were excluded.

The search was not restricted by language or time period, in order to reflect the current state of knowledge as broadly as possible. Search strategy was also validated on previously known relevant papers identified during the initial literature review and literature review. The final search was conducted on 28 February 2026.

To maintain the conceptual focus of this scoping review, eligible studies were required to analyze an NSD classification, morphological deviation category, severity grade, or standardized NSD assessment method as the primary exposure or stratification variable in relation to quantitative craniofacial, maxillary, mandibular, palatal, dentofacial, or asymmetry-related morphometric outcomes. Studies were excluded when NSD was reported only as presence or prevalence within predefined malocclusion, sinonasal, airway, or disease groups, when NSD was assessed only as a secondary sinonasal finding, when no quantitative craniofacial or dentofacial morphometric outcome was analyzed in relation to the NSD assessment method or when postoperative or treatment-induced changes in NSD were evaluated.

The Mladina classification was considered one of the predefined NSD classification systems of interest, but it was not used as the sole eligibility criterion.

### 2.1. Search Strategy and Information Sources

The search strategy was developed to identify as broadly as possible publications concerning the relationship between NSD and craniofacial morphology. Its basic structure included terms related to NSD and terms describing craniofacial morphology, facial asymmetry, maxilla, mandible, palate, and malocclusion.

The queries were then individually tailored to the specifics of each database and search engine, taking into account their different syntactic requirements and search capabilities. The search was conducted in the following databases and search engines: Bielefeld Academic Search Engine (BASE; over 400,000,000 records), The Cochrane Library (over 2,000,000 records), Embase (over 50,000,000 records), PubMed (over 40,000,000 citations), and Google Scholar (over 600,000,000 records). In order to obtain the most complete picture of the available literature, no filters were used to limit search results by language, publication date, or document type. Google Scholar searches were conducted using the “allintitle:” operator to increase the specificity and reproducibility of results. This limited the search to records containing key terms in the title and reduced the number of results that were not directly related to the topic.

Specific queries for each engine and database can be found in Table A1 in Appendix A.

### 2.2. Selection Process

Identified records were first manually deduplicated using the Rayyan tool (version 2026-03-01, Qatar Computing Research Institute, Doha, Qatar and Rayyan Systems, Cambridge, MA, USA). Two independent, blinded researchers (J.W. and M.K.) then performed an initial assessment of publication eligibility based on titles and abstracts. In case of discrepancies, the record was forwarded to the full-text review stage. Full-text analysis was also independently conducted by the same two researchers (J.W. and M.K.), and in cases of disagreement, the final decision was made by a third researcher (F.B.).

### 2.3. Data Collection Process

Data extraction was performed independently by two investigators (J.W. and M.K.) based on full-text analysis of included studies. Primary outcomes were quantitative skeletal, asymmetry, or dentofacial measures reported in association with NSD. These included maxillary, mandibular, palatal, transverse craniofacial, facial asymmetry, malocclusion, and nasal volume parameters. Secondary outcomes included the method used to assess NSD, such as the Mladina classification, other classifications, angular or linear measurements, volumetric assessment, or imaging-based severity assessment. The direction of the reported association between NSD and each extracted morphological outcome was also recorded. Additional data extracted from eligible publications included author, year of publication, country, study design, sample size, age of participants, study setting, imaging method, inclusion and exclusion criteria, comparison groups, statistical methods, main findings, and limitations reported by the original authors. In case of discrepancy between the two investigators, a third investigator (F.B.) had the casting voice.

### 2.4. Data Items

The extracted data were categorized into the following: study characteristics, NSD assessment method, craniofacial outcome domain, and direction of the reported association. Craniofacial outcome domains included palatal/transverse maxillary morphology, local craniofacial asymmetry, global facial asymmetry, malocclusion or skeletal pattern, basic maxillomandibular dimensions, and nasal volume. These domains were then used to generate a descriptive synthesis, tables, and an evidence map.

### 2.5. Critical Appraisal

Formal critical appraisal and certainty-of-evidence assessment were not performed, consistent with the purpose of a scoping review, which was to map the available evidence rather than evaluate intervention effects or estimate pooled outcomes.

### 2.6. Synthesis Methods

Due to the heterogeneity of study designs, NSD assessment methods, imaging protocols, and craniofacial outcome measures, quantitative synthesis was not performed. Results were synthesized descriptively and presented in comparative tables.

## 3. Results

### 3.1. Study Selection

**Figure 1:**
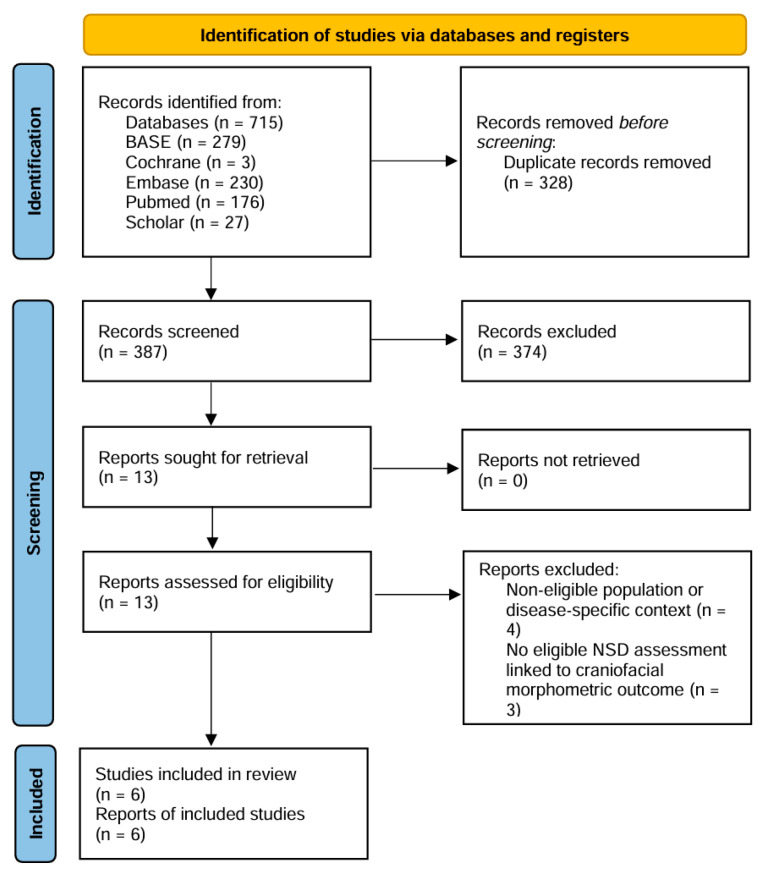
PRISMA flow diagram.

A total of 715 records were identified, of which 387 publications remained for further review after removing duplicates. After reviewing titles and abstracts and then assessing the full texts, 6 studies that met all established inclusion criteria were ultimately included in the review (Figure 1). The significant number of excluded records was due to the broadly designed search strategy, which aimed to capture as much of the available literature on the topic as possible. The evaluation of the papers was carried out in a very detailed manner, the inclusion criteria were very narrow, hence the relatively small number of articles ultimately qualified for the study.

### 3.2. Characteristics of the Included Studies and Results of Individual Studies

The tables below present characteristics of the included studies (Table 2, Table 3, Table 4 and Table 5). This is a descriptive presentation that reflects the nature of the scoping review.

### 3.3. Results of Syntheses

Six studies assessing the relationship between NSD and selected craniofacial morphology were included in the final analysis. Sample sizes varied, ranging from 40 to 337 participants. Most studies were observational, cross-sectional, or retrospective, and the dominant imaging methods were computed tomography (CT) and cone-beam computed tomography (CBCT). One study additionally used three-dimensional geometric morphometry. Detailed study characteristics are presented in Table 1.

**Figure 2:**
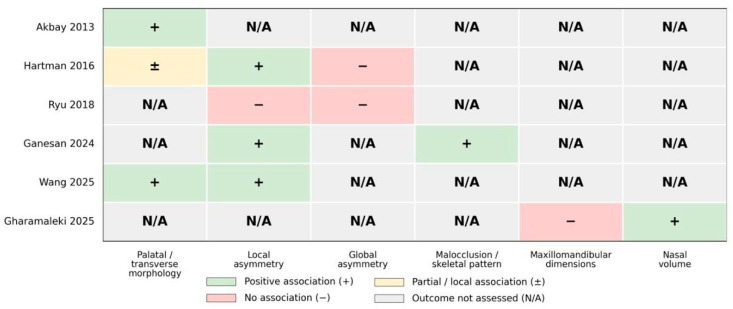
Evidence map of reported associations between NSD and craniofacial outcomes (created by F.B.) [12,14,15,16,22,23].

The included studies varied in study populations and in the craniofacial outcome domains assessed. Some authors focused on transverse maxillary and palate morphology, others analyzed global or local craniofacial asymmetry, and still others assessed the relationship between NSD and malocclusion type, maxillomandibular dimensions, or nasal volume. This variability in the scope of the analyzed outcome measures is reflected in both Table 3 and Figure 2.

#### 3.3.1. Heterogeneity of NSD Assessment Methods

**Figure 3:**
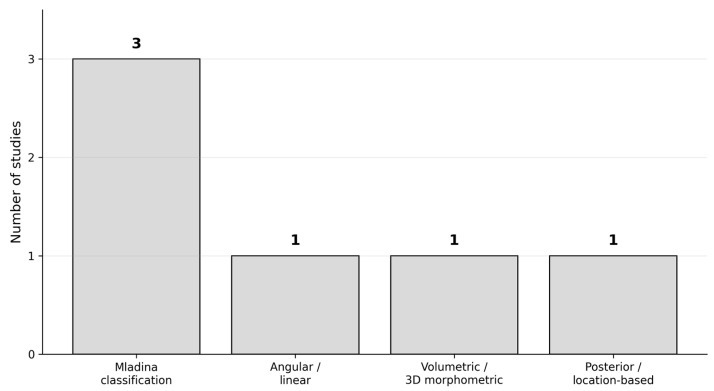
Methods used to assess NSD across the included studies (created by F.B.).

**Figure 4:**
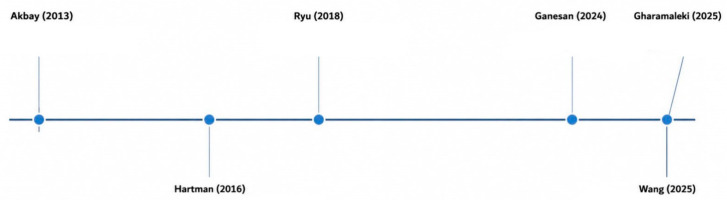
Timeline of included studies (created by F.B.) [12,14,15,16,22,23].

Methodological analysis revealed significant heterogeneity in the assessment of NSD. Three studies used the Mladina classification, two of which related it to occlusal-skeletal features or asymmetry, and one to basic maxillomandibular dimensions and nasal volume. Wang’s study employed a quantitative approach based on the SDA, DSW, DSCA, and SVL parameters, allowing for the analysis of the relationship between the severity of NSD and the lateral asymmetry of craniofacial structures [12]. Hartman treated NSD as a continuous variable and assessed it using three-dimensional volumetric analysis, comparing septal volume with a model of an undeviated septum [15]. Akbay used a classification based on the location and morphology of posterior septal deviation, distinguishing groups with convex deviation, crest/spine deviation, and without NSD [22]. These differences are summarized in Table 2 and presented schematically in Figure 3. From a methodological perspective, this means that individual studies did not use the same measurement construct, even if they all addressed NSD. In practice, this makes direct comparison of results difficult and limits the ability to draw simple, uniform conclusions for the entire group of studies. The timeline of included studies is presented in Figure 4.

#### 3.3.2. NSD and Palatal or Transverse Maxillary Morphology

The most consistent pattern of results concerned the relationship between NSD and local changes within the nasomaxillary complex, particularly the transverse morphology of the maxilla and palate. Akbay demonstrated a significant positive correlation between posterior septal deviation and the depth of the palatomaxillary arch, reporting a strong positive relationship between posterior septum deviation and the depth of the maxillopalatal arch [22]. Wang confirmed significant associations between NSD parameters and transverse asymmetry of the maxilla, alveolus, and cranial base, demonstrating differences between subgroups with varying degrees of SDA, DSW, and DSCA [12].

Both studies, despite differing methodology for assessing NSD, indicated that septal deviation may be specifically related to local transverse changes and not necessarily to the overall growth pattern of the entire facial skeleton. This pattern is also clearly visible in Figure 2, where the palatal/transverse morphology domain is among the best supported by available research.

#### 3.3.3. NSD and Facial Asymmetry: Global Versus Local Relationships

Regarding facial asymmetry, the results were less clear. Hartman found no relationship between NSD and the global extent of asymmetry, but found local correlations, primarily in the nasal floor and palate [15]. Ryu, based on 3D CBCT and the Maeda Asymmetry Index, also failed to demonstrate a statistically significant correlation between the degree of NSD and facial asymmetry, nor between NSD and individual craniofacial points [23]. Ganesan, on the other hand, demonstrated that higher degrees of NSD were associated with greater severity of ANS and menton deviation and greater facial asymmetry [14]. This indicates that the lack of consensus primarily concerns global asymmetry, while some studies still suggest the possibility of capturing relationships in more narrowly defined local domains or using more clinically focused cephalometric measurements. This distribution of results is reflected in Table 3 and Table 4.

#### 3.3.4. NSD vs. Malocclusion Type

The relationship between NSD and malocclusion type has been directly assessed in only one study. Ganesan demonstrated a statistically significant association between the degree of septal deviation and the type of malocclusion, with Class II Division 1 predominating and Class III features more frequently occurring with higher degrees of NSD [14]. The authors also indicated that maxillary and mandibular asymmetry and facial deviations increased with increasing degrees of NSD. However, since only one study directly examined this domain, it should be considered underrepresented in the current literature. From a clinical perspective, however, this is an important direction, as it links the assessment of NSD with more directly observable orthodontic consequences. This is included in Table 4 as a domain with a limited but positive evidence base.

#### 3.3.5. NSD and Basic Maxillomandibular Dimensions

A different picture was obtained in the Gharamaleki study, which, with the largest sample size, found no significant differences in basic maxillomandibular parameters between patients with and without NSD [16]. No significant differences were found for the ANS-PNS distance, maxillary width, maxillary height, maxillary nasal base height, or mandibular arch width measured at the level of the first molars. At the same time, the authors demonstrated an increase in nasal volume in the presence of NSD.

This result suggests that the potential impact of NSD may not be detectable in simple linear measurements of basic maxillomandibular dimensions or that the impact may involve more subtle, local, and spatial patterns of asymmetry than overall skeletal dimensions. Therefore, at the level of the overall evidence map, this domain remains ambiguous.

#### 3.3.6. Overall Pattern of the Mapped Evidence

The overall analysis indicates that the most consistent findings relate to local changes in the palate and maxillary transverse morphology, whereas data relating to global facial asymmetry and basic maxillomandibular dimensions remain inconsistent. Figure 2 shows that positive studies predominate in the domains of local morphology and transverse asymmetry, whereas negative or equivocal results predominate in global facial asymmetry. Table 4 synthesizes this picture as a framework for the research field, not as a hierarchy of evidence strength.

## 4. Discussion

### 4.1. Principal Findings

This scoping review demonstrates that the relationship between NSD and craniofacial morphology is neither uniform nor easily captured by a single method of analysis. The most important finding from mapping the available studies is that NSD is more often associated with local or transverse morphological changes within the nasomaxillary complex than with global asymmetry across the entire face [12,15,22]. In practice, this means that studies focusing on the palate, maxilla, and adjacent structures yield more positive results than studies assessing craniofacial asymmetry as a global construct [12,15,22].

This pattern is significant not only from a cognitive but also from a methodological perspective. The nasal septum is a central structure, but its potential growth effect does not necessarily manifest as a uniform deformation of the entire facial skeleton. Any associations are much more likely to involve adjacent, more sensitive anatomical areas, such as the palate, nasal floor, or transverse maxillary development. In this sense, the results of Akbay, Hartman, and Wang are more consistent with each other than might appear at a superficial reading, as they all point to a local dimension of the NSD–morphology relationship, albeit using different measurement tools [24,25,26].

### 4.2. Why Are the Findings Inconsistent?

The most likely source of discrepancies between the results of the included studies is methodological heterogeneity. Not only did the researchers evaluate different populations and different endpoints, but above all, they used different definitions of NSD itself [14,15,16,22]. In some studies, the septum was categorically defined according to the Mladina classification, in others using angular and linear parameters, and in still others as a continuous variable based on 3D volumetric analysis. Such differences are not merely technical; they directly influence which aspect of NSD is actually measured [9,15,27].

An additional source of inconsistency is the range of outcome measures analyzed. Gharamaleki did not confirm the association of NSD with basic maxillomandibular dimensions, but his study was not designed to capture subtle, local patterns of asymmetry to the same extent as the 3D analyses by Wang or Hartman [12,15,16]. Similarly, the lack of association with global facial asymmetry in Ryu study does not necessarily mean that NSD is clinically indifferent, but rather that its influence may be too local or too dependent on the anatomical domain to be revealed in a generalized asymmetry index [23].

### 4.3. The Role of the Mladina Classification

Of particular note is the Mladina classification, which was the most commonly used categorical system in the included studies. Its value lies in its anatomical clarity and broad clinical application, particularly in otorhinolaryngology. However, the results of studies using the same system were inconsistent. Ganesan demonstrated an association between higher degrees of NSD and greater asymmetry and a different distribution of malocclusion types, while Gharamaleki did not confirm the effect of NSD on basic maxillomandibular parameters [14,16].

This suggests that morphological classification of the septum alone is insufficient to predict a specific pattern of skeletal abnormalities. It may be useful as a tool for organizing the NSD phenotype, but its prognostic value with respect to craniofacial morphology remains limited unless accompanied by spatial analysis of more precise variables. For future research, this suggests the need to combine categorical classifications with quantitative and three-dimensional measurements [9,14,16].

### 4.4. Clinical Interpretation

From a clinical perspective, the results of this review do not support assigning NSD as a clear, stand-alone causative factor for global facial asymmetry or generalized maxillary malformations. A more cautious approach is to consider NSD as a significant component of the clinical picture in patients with lateral asymmetry, altered palate morphology, or a specific pattern of malocclusion, but its significance should be assessed contextually. This interpretation is consistent with available research and is methodologically much safer than making causal claims [15,16,28].

In orthodontic and surgical practice, this means that the presence of NSD can be considered part of a broader patient evaluation, particularly when lateral maxillary malformations, local asymmetry, or signs of chronic nasal obstruction are present. However, this does not mean that every patient with NSD will exhibit a specific pattern of craniofacial deformity [12,14,26,28].

### 4.5. NSD, Craniofacial Morphology and Sinonasal Conditions

It is worth noting that several studies have addressed NSD and craniofacial morphological changes in the context of sinonasal conditions, including odontogenic sinusitis [29]. However, the present manuscript was not intended to focus on disease-related sinonasal conditions, but rather on NSD as an anatomical finding in relation to craniofacial morphology. Nevertheless, prolonged local inflammation, particularly during growth, may influence craniofacial anatomy, and clinicians should keep this potential relationship in mind.

## 5. Limitations

This review has several significant limitations that should be considered when interpreting the results, for example, differences in sample sizes, patient age, inclusion criteria, imaging modalities and the way NSD was described and graded.

First, the number of studies meeting the inclusion criteria was small, and their study designs were significantly heterogeneous. This limits the ability to draw broad generalizations and means that the resulting picture should be considered primarily as a map of research directions rather than a basis for strong causal statements.

Second, the included studies differed in both the way they defined NSD and the type of outcome measures analyzed. The Mladina classification, angular parameters, assessment of posterior curve location, and 3D volumetric analysis do not precisely describe the same measurement construct. At the same time, the endpoints examined covered a very wide spectrum—from palatal depth and facial asymmetry to malocclusion and nasal volume. It is also worth mentioning that there were differences in imaging protocols between included studies. This heterogeneity limits the synthetic interpretation of the results.

Third, the majority of the included data came from observational studies of a cross-sectional or retrospective nature. Such designs allow for the assessment of co-occurrence of features but do not allow for causal inference. Therefore, even significant associations between NSD and selected craniofacial parameters cannot be interpreted as evidence of a direct effect of NSD on facial skeletal development.

Fourth, in the case of Ryu, a working paper based on a translation of the abstract and basic methodological data was used, rather than a complete, native extraction from the published full-text article. Although this material was sufficient to map the direction of the results, the level of detail in this study was less than in the other studies [23].

Fifth, this scoping review did not conduct a formal assessment of methodological quality or level of evidence. This was consistent with the review’s goal of comprehensively mapping the available literature, rather than hierarchically assessing the strength of evidence. This means that the presented results should be interpreted as a description of the structure and scope of the research field, not as a ranking of the credibility of individual conclusions.

Summing it up, this scoping review should be seen as a map of existing evidence and not as a basis for definitive causal inference.

## 6. Conclusions

NSD should not currently be interpreted as a uniform marker of global craniofacial morphological abnormalities. The available evidence suggests that NSD assessment methods, including the Mladina classification and other standardized quantitative approaches, may be associated with selected local or transverse features of the nasomaxillary complex, particularly within the palate, nasal floor, maxilla, and adjacent dentoalveolar structures. However, the evidence remains limited, heterogeneous, and inconsistent across outcome domains, especially with regard to global facial asymmetry, basic maxillomandibular dimensions, and malocclusion patterns.

Because the included studies differed substantially in NSD classification or measurement methods, imaging protocols, populations, and craniofacial outcomes, the findings should be interpreted as a map of the available evidence rather than as proof of causality. Current data do not allow for the determination of whether NSD directly contributes to craniofacial development or merely co-occurs with selected morphological patterns. Future studies should combine standardized NSD classifications, particularly the Mladina system, with precise three-dimensional craniofacial assessment in more homogeneous populations. Such an approach may help clarify which specific NSD phenotypes are associated with defined local or transverse craniofacial features and whether these associations have developmental or clinical significance.

## Figures and Tables

**Table 1:** Eligibility criteria.

Domain	Inclusion Criteria	Exclusion Criteria
Population	Patients with diagnosed NSD not caused by sinonasal conditions, irrespective of age or treatment status.	Studies limited to sinonasal conditions, syndromic craniofacial conditions, isolated cleft populations, or non-human subjects.
Concept	Studies assessing NSD using a defined classification system, morphological deviation category, severity grade, or standardized assessment method and analyzing its relationship with quantitative craniofacial, maxillary, mandibular, palatal, dentofacial, or asymmetry-related morphometric outcomes.	Studies reporting only NSD presence/prevalence, ear/nose/throat symptoms, sinonasal findings, airway outcomes, or NSD distribution within predefined disease or malocclusion groups without eligible quantitative craniofacial or dentofacial morphometric outcomes analyzed in relation to NSD assessment.
Context	Clinical or academic settings using radiographic imaging (CBCT, CT, or cephalometry) for morphometric analysis.	Experimental, simulation-based, or purely laboratory studies without clinical imaging data.
Comparators	Any comparator group or absence of comparator.	Not applicable.
Outcomes	Any reported quantitative skeletal, asymmetry, or dentofacial measurement associated with NSD.	Studies reporting only subjective clinical findings without measurable outcomes.
Study design	Original clinical or academic observational studies reporting extractable quantitative data relevant to the review question, including cross-sectional, cohort, case–control, retrospective, comparative, and imaging-based morphometric studies.	Case reports, narrative reviews, editorials, letters, opinion papers, conference abstracts without accessible full text, and studies without extractable quantitative data.
Timeframe	No restriction on publication date.	Not applicable.
Language	All languages with accessible full text.	Studies without retrievable full text.

**Table 2:** Characteristics of included studies.

Study	Setting	Sample Size	Age	Study Design	Imaging/Assessment Methods	Main Craniofacial Outcomes Assessed
Akbay et al., 2013 [22]	Turkey; university hospital database	150 CT scans, 3 groups of 50 each	Adults ≥ 18 years; mean age by groups: 28.02 ± 10.43, 34.70 ± 18.21, 37.02 ± 16.07 years	Retrospective CT-based comparative study	Paranasal sinus CT in coronal plane; NSD grouped as convex deviation, crest/spur deviation, or no deviation; measured PIL, PAD, MPAA, SVL, DSL, DSCA, PAD/PIL	Depth and shape of maxillopalatal arch in relation to posterior septal deviation
Hartman et al., 2016 [15]	USA; geographically diverse adult sample from Iowa-based group	55 adults	19–70 years in European-derived subsample; 20–67 years in African-derived subsample	Observational CT-based morphometric study	CT with 3D geometric morphometrics; NSD quantified volumetrically as septal volume relative to modeled non-deviated septum; 41 craniofacial landmarks analyzed with Procrustes methods	Global facial asymmetry and localized asymmetry in nasal, palatal, and lateral facial regions
Wang et al., 2025 [12]	China; hospital of stomatology archive, 2020–2023	114 subjects	Adults > 18 years	Retrospective cross-sectional study	CBCT; NSD quantified with SDA, DSW, DSCA, SVL; craniofacial transverse measures of maxilla, dentoalveolus, and cranium; subgrouping by NSD severity for SDA, DSW, DSCA	Transverse craniofacial asymmetry of maxilla, dentoalveolus, cranial base, zygomatic, orbital, palatal, molar, TMJ and related structures
Gharamaleki et al., 2025 [16]	Türkiye; Biruni University Hospital CT database	337 patients	Mean 34.9 ± 10.95 years; inclusion > 16 years	Retrospective CT study	CT; NSD classified using Mladina system; measurements: ANS-PNS length, maxillary width, maxillary height, maxillary depth/nasal base height, mandibular molar width, nasal volume	Maxillomandibular morphology and nasal volume in relation to NSD presence/type
Ganesan et al., 2024 [14]	India; two-center study (orthodontics + ENT)	40 patients	18–25 years	Observational cross-sectional study	Clinical ENT exam + CT-PNS with Mladina classification; orthodontic evaluation with lateral cephalogram, postero-anterior cephalogram, facial photographs, study models	Malocclusion type, skeletal pattern, ANS/menton deviation, facial asymmetry and cephalometric characteristics relative to NSD grade
Ryu et al., 2018 [23]	South Korea; Ajou University Dental Hospital	92 patients	Adults ≥ 19 years; mean age not reported.	CBCT-based observational study	CBCT 3D; Mladina classification; 3D craniofacial landmarks analyzed with Maeda asymmetry index; t-test and Pearson correlation	Facial asymmetry in relation to NSD degree/type

**Table 3:** Methods used for NSD assessment across included studies.

Study	NSD Definition/Classification	Quantitative NSD Parameters	Notes on NSD Assessment
Akbay et al., 2013 [22]	Deviation categorized into convex posterior deviation, crest/spur deviation, and no deviation	DSL, DSCA, SVL measured on coronal CT; posterior septal relationships analyzed against palatal measures	Study focused specifically on posterior septal deviation and its morphologic relationship with the maxillopalatal arch
Hartman et al., 2016 [15]	No categorical class; NSD treated as a continuous volumetric 3D variable	Septal deviation calculated as percentage of nasal septal volume relative to modeled non-deviated septal volume; 100% = no deviation, >100% = deviation	This was the most explicitly volumetric/morphometric NSD approach among included studies
Wang et al., 2025 [12]	No named categorical system; NSD severity stratified by parameter thresholds	SDA, DSW, DSCA, SVL; severity groups defined for SDA, DSW, and DSCA	Mild/moderate/severe groups: SDA ≤ 9°, 9–15°, ≥15°; DSW < 5.08 mm, 5.08–7.38 mm, ≥7.38 mm; DSCA > 155°, 148–155°, ≤148°
Gharamaleki et al., 2025 [16]	Mladina classification (Types I–VII)	NSD presence vs. absence; ANOVA across Mladina types	Distribution reported: no NSD 13.1%; Type I 21.1%; II 10.7%; III 16.6%; IV 7.4%; V 10.1%; VI 14.8%; VII 6.2%
Ganesan et al., 2024 [14]	Mladina classification used by radiologists on CT-PNS	Grading-based clinical and cephalometric comparisons; exact full grade distribution not visible in provided excerpt	Patients with grade I NSD were excluded according to methods section; only grades II–VII were assessed
Ryu et al., 2018 [23]	Mladina classification	Degree of septal deviation also quantified angularly; correlation tested against 3D asymmetry indices	In the translated summary, NSD prevalence was 58% and type 2 was most common

**Table 4:** Direction of evidence by outcome domain.

Study	Palatal/Transverse Maxillary Morphology	Local Craniofacial Asymmetry	Global Facial Asymmetry	Malocclusion/Skeletal Pattern	Basic Maxillomandibular Dimensions	Nasal Volume
Akbay et al., 2013 [22]	Positive association: posterior septal deviation correlated with maxillopalatal depth; r = 0.479, *p* = 0.001	Not primary endpoint	Not assessed as global construct	Not assessed	Not assessed	Not assessed
Hartman et al., 2016 [15]	Local association in palatal region	Yes: associated primarily with nasal floor and palatal region asymmetry	No: no correlation with overall magnitude of asymmetry	Not assessed	Not assessed	Not assessed
Wang et al., 2025 [12]	Positive association: NSD variables significantly associated with transverse asymmetry of maxilla and dentoalveolus	Yes: for transverse asymmetry across multiple structures	Not framed as global whole-face asymmetry in abstracted text	Not assessed	Not primary outcome	Not assessed
Gharamaleki et al., 2025 [16]	No significant differences in measured maxillary parameters between NSD and no-NSD groups	Not primary endpoint	Not assessed	Not assessed	No clear association with ANS-PNS, maxillary width, maxillary height, maxillary nasal base height, or mandibular molar width	Positive association: nasal volume increased in presence of NSD, *p* = 0.01
Ganesan et al., 2024 [14]	Not primary transverse maxillary study	Positive association: greater NSD grade associated with greater ANS/menton deviation and asymmetry	Not reported as separate global asymmetry metric	Positive association: malocclusion distribution differed by NSD grade; *p* = 0.006; Class II div 1 most common; Class III more common in higher NSD grades	Not primary endpoint	Not assessed
Ryu et al., 2018 [23]	Not primary endpoint in provided translation	No significant associations identified for craniofacial landmarks	No: no significant correlation between NSD degree and facial asymmetry	Not assessed	Not assessed	Not assessed

**Table 5:** Concise synthesis of the mapped evidence.

Domain	Overall Pattern Across Included Studies	Supporting Studies
Palatal/transverse morphology	Findings generally support an association between NSD and localized transverse or palatal changes, but not uniformly across all studies	Akbay 2013 [22]; Wang 2025 positive [12]
Global facial asymmetry	Evidence does not consistently support a relationship between NSD and global facial asymmetry	Hartman 2016 no global association [15]; Ryu 2018 no significant correlation [23]
Localized asymmetry	Some studies support a relationship, especially in palatal/nasal-floor or transverse craniofacial regions	Hartman 2016 [15]; Wang 2025 [12]; Ganesan 2024 [14]
Malocclusion/skeletal class	Limited but positive evidence suggests higher NSD severity may be associated with malocclusion pattern and asymmetry	Ganesan 2024 [14]
Basic maxillomandibular dimensions	Evidence is inconsistent; one larger Mladina-based CT study did not find significant differences in core dimensions	Gharamaleki 2025 [16]
Nasal volume	Reported as increased in the presence of NSD in one included study	Gharamaleki 2025 [16]

## Data Availability

The original contributions presented in this study are included in the article. Further inquiries can be directed to the corresponding author.

## Supplementary Materials

The following supporting information can be downloaded at: https://www.mdpi.com/article/10.3390/jcm15134853/s1, Table S1: PRISMA 2020 Checklist.

## Abbreviations

The following abbreviations are used in this manuscript:

ANOVA	Analysis of Variance
ANS	Anterior Nasal Spine
ANS-PNS	Anterior Nasal Spine–Posterior Nasal Spine
BASE	Bielefeld Academic Search Engine
CBCT	Cone-Beam Computed Tomography
CT	Computed Tomography
CT-PNS	Computed Tomography of the Paranasal Sinuses
DSL	Deviated Septal Length
DSCA	Deviated Septal Curve Angle
DSW	Deviated Septal Width
ENT	Ear, Nose, and Throat
JBI	Joanna Briggs Institute
MPAA	Maxillopalatal Arch Angle
NSD	Nasal Septal Deviation
OSF	Open Science Framework
PAD	Palatal Arch Depth
PAD/PIL	Palatal Arch Depth/Palatal Interalveolar Length Ratio
PCC	Population, Concept, Context
PIL	Palatal Interalveolar Length
PRISMA-ScR	Preferred Reporting Items for Systematic Reviews and Meta-Analyses Extension for Scoping Reviews
SDA	Septal Deviation Angle
SVL	Septal Vertical Length
TMJ	Temporomandibular Joint

## Appendix A

**Table A1:** Specific queries for each database.

Database	Specific Query
BASE	(“nasal septum deviation” OR “septal deviation” OR “deviated nasal septum”) AND (craniofacial OR malocclusion OR “skeletal class” OR “facial skeleton” OR maxillofacial OR asymmetry) AND (cephalometry OR CBCT OR “cone beam” OR CT OR imaging)
Cochrane	(“nasal septum deviation” OR “septal deviation” OR “deviated nasal septum”) AND (craniofacial OR malocclusion OR “skeletal class” OR “facial skeleton” OR maxillofacial OR asymmetry)
Embase	(“nasal septum deviation” OR “septal deviation” OR “deviated nasal septum”) AND (craniofacial OR malocclusion OR “skeletal class” OR “facial skeleton” OR maxillofacial OR asymmetry) AND (cephalometry OR CBCT OR “cone beam” OR CT OR imaging)
PubMed	(“Nasal Septum”[Mesh] OR “Nasal Septum/abnormalities”[Mesh] OR “nasal septum deviation”[tiab] OR “nasal septal deviation”[tiab] OR “deviated nasal septum”[tiab] OR “septal deviation”[tiab]) AND (malocclusion[Mesh] OR “Jaw Abnormalities”[Mesh] OR craniofacial[tiab] OR “facial skeleton”[tiab] OR maxillofacial[tiab] OR malocclusion*[tiab] OR “skeletal class”[tiab] OR “Class II”[tiab] OR “Class III”[tiab] OR asymmetr*[tiab]) AND (cephalometr*[tiab] OR “lateral cephalogram”[tiab] OR CBCT[tiab] OR “cone beam computed tomography”[tiab] OR CT[tiab] OR imaging[tiab])
Scholar	allintitle: (“nasal septum deviation” OR “septal deviation” OR “deviated nasal septum”) AND (craniofacial OR malocclusion OR “skeletal class” OR “facial skeleton” OR maxillofacial OR asymmetry)
